# OTUB1 Promotes Odontogenesis in Dental Pulp Stem Cells by Mediating TPM1 Deubiquitination and Activating Autophagy

**DOI:** 10.1016/j.identj.2026.109814

**Published:** 2026-08-06

**Authors:** Tingjie Gu, Wei Song, Yifan Pan, Jie Yi, Tao Xu, Kai Xu, Zhengmei Qian, Dandan Song, Rui Gu, Qiang Chen

**Affiliations:** a Yancheng Stomatological Hospital, Yancheng City, Jiangsu Province, 224000, China; b Xuzhou Medical University, Xuzhou, Jiangsu, China; c Department of Stomatology, Nanjing Central Hospital, Nanjing, Jiangsu, China

**Keywords:** DPSCs, Odontogenic differentiation, Deubiquitination, Autophagy

## Abstract

**Objectives:**

DPSCs, known for their robust multipotent differentiation, are top candidates for engineering dental pulp tissues. OTUB1 is a critical deubiquitinating enzyme that regulates multiple biological processes and disease contexts. In our study, the function of OTUB1 in regulating the odontogenesis of DPSCs was investigated.

**Materials and methods:**

The verification of phenotypes involved experiments with gain and loss of function, along with a rat tooth pulp model. Proteomic analysis combined with LC-MS/MS analysis was conducted to identify the target in the process of OTUB1 mediated-deubiqutination in DPSCs.

**Results:**

Odontogenic differentiation capacities were influenced by the increase or decrease of OTUB1. Increased levels of OTUB1 promote dentin formation in a rat tooth pulp model. Moreover, OTUB1 directly interacts with TPM1. By deubiquitinating TPM1, OTUB1 prevents its proteasomal degradation, thereby upregulating TPM1 protein levels and increasing autophagy in DPSCs. Consequently, the beneficial impact on odontogenic differentiation due to OTUB1 overexpression was nullified by the targeted removal of TPM1 or the inhibition of autophagy in DPSCs.

**Conclusions:**

The OTUB1-mediated deubiquitination/TPM1/autophagy pathway promotes odontogenic differentiation in DPSCs, thereby presenting novel strategies for enhancing dentin regeneration.

## Introduction

Pulpitis and periapical periodontitis are common clinical conditions characterized by inflammation of the dental pulp or periapical tissues, often manifesting as tooth pain and periapical swelling. Currently, root canal therapy is employed as the mainstay treatment.^1^ However, the removal of the pulp compromises the tooth's natural biological defenses, thereby increasing the risk of severe caries and apical periodontitis.^2^ Numerous studies have examined the extent to which the presence of dental pulp significantly influences the prognosis of a treated tooth.^3^, ^4^, ^5^

Mesenchymal stem cell (MSC)-based tissue regeneration has attracted substantial attention in contemporary medical research.^6^^,^^7^ Dental pulp stem cells (DPSCs), a subset of MSCs sourced from dental tissues, are recognized as promising candidates for regenerative applications due to their remarkable proliferative capabilities and potential for multilineage differentiation.^8^^,^^9^ Studies have shown that the committed differentiation of DPSCs is a meticulously regulated process, orchestrated by a complex biological network.^10^, ^11^, ^12^

Ubiquitination represents a fundamental post-translational modification that plays a crucial role in the regulation of various biological processes. This modification involves covalently attaching ubiquitin molecules to substrate proteins, resulting in their degradation or alteration in function.^13^^,^^14^ This system is regulated by two opposing enzyme groups: the ubiquitinating enzymes (E1, E2, and E3), which catalyze the attachment of ubiquitin chains,^14^^,^^15^ and deubiquitinases (DUBs), which hydrolyze these linkages to edit signaling outcomes or prevent protein degradation.^16^ Among DUBs, Otubain1 (OTUB1), which belongs to OTU domain family, has been identified as an important regulatory protein.^17^ It fine-tunes crucial pathways in cancer, DNA damage, and neurodegeneration.^18^, ^19^, ^20^, ^21^ Extending its functional repertoire, Zhu et al recently identified a role for OTUB1 in osteoblastic bone formation.^22^ Nevertheless, how OTUB1 and its deubiquitination function influence the committed differentiation of DPSCs remains to be further fully characterized.

This study utilizes a comprehensive methodology, integrating bioinformatics analysis with animal models, to elucidate the pivotal function of OTUB1 in the odontogenic differentiation of DPSCs. Our research uncovers a novel mechanism whereby OTUB1 promotes the deubiquitination of TPM1, thereby initiating autophagy and augmenting differentiation.

## Materials and methods

### Cell isolation and culture

The accurate approach of isolation and culture of DPSCs was conducted in accordance with an established protocol^23^ and received approval from the Ethics Committee of Yancheng Stomatological Hospital (PJ2024-022-01). In brief, the third molars were obtained from healthy young donors, stored in PBS, and promptly delivered to the laboratory under sterile conditions for subsequent cell isolation. After repeated cleaning with PBS and α-MEM**,** the teeth were carefully cracked open, and the pulp tissue was extracted and rinsed with PBS containing 1% penicillin/streptomycin. The harvested tissue was then minced into small fragments and enzymatically digested at 37°C for 20 minutes using a solution of 3 mg/mL type I collagenase and 4 mg/mL dispase (Sigma, USA). The isolated cells were plated and maintained in α-MEM containing 10% fetal bovine serum (FBS) and 1% penicillin/streptomycin, and incubated at 37 °C under 5% CO₂ upon digestion termination. Once the cell density reached 80% to 90%, the cells were digested and subcultured at a 1:3 ratio, with the medium being changed every two days.

### Trilineage differentiation of DPSCs

For odontogenic differentiation, the basal culture medium was enriched with 10 nM dexamethasone, 100 μM ascorbic acid, and 2 mM β-glycerophosphate (Sigma, USA) to produce the induction medium. Following a 14-day induction period, DPSCs were fixed in 4% PFA for 30 minutes and subsequently stained with Alizarin Red S (ARS, Oricell, China) for 30 minutes. Semi-quantitative analysis was performed by eluting the bound dye with 10% cetylpyridinium chloride (CPC), and the measured calcium levels were normalized against the total protein content of each sample.

Adipogenic differentiation was induced via a commercial kit (Oricell, China). Upon reaching near-complete confluence (90%-100%), DPSCs were treated alternately with the adipogenic induction medium A and B solutions as per the manufacturer’s protocol. Lipid droplets were detected by Oil Red O staining at approximately 3 weeks post-induction.

Chondrogenic differentiation was assessed via pellet culture. DPSCs were aggregated in a 15 mL conical tube and maintained in chondrogenic induction medium (Oricell, China). Once cell pellets had formed to an appropriate size, they were fixed, embedded in OCT compound, and sectioned into 5 μm slices for histological analysis by Alcian blue staining.

### Cell transfection and lentivirus infection

Specific small interfering RNA (siRNA) sequences targeting OTUB1 and TPM1 were commercially synthesized by Kileafbio Co. Ltd., as detailed in "Supplementry Table 1. Transfection of DPSCs was performed at approximately 70% confluence using the Kileafbio CP transfection kit. To achieve stable overexpression, DPSCs were transduced with recombinant lentiviruses (GenePharma, China) encoding either the full-length OTUB1 or a non-targeting control (NC). The cells were cultured in α-MEM supplemented with 10% fetal bovine serum (FBS) and 10 μg/mL polybrene to enhance transduction efficiency.

### Quantitative real-time polymerase chain reaction (qRT‒PCR)

After extracting total RNA from DPSCs using TRIzol reagent, the RNA was reverse-transcribed into complementary DNA (cDNA) utilizing the PrimeScript RT kit (Vazyme, China). Quantitative real-time PCR (qRT-PCR) was then performed using the ChamQ Universal SYBR Green qPCR Master Mix (Vazyme, China) on a qTOWER3/G PCR system. The expression levels of genes associated with odontogenesis were quantified using the 2−ΔΔCt method, with GAPDH serving as the endogenous control. Detailed primer sequences used for qRT-PCR are provided in Supplementary Table 2.

### Western blotting and co-immunoprecipitation (CO-IP)

Proteins were extracted from DPSCs using RIPA lysis buffer (Beyotime, China) containing PMSF protease inhibitor on ice. We use a BCA assay kit (Beyotime, China) to determine protein concentrations. Following SDS-PAGE separation, proteins were transferred onto PVDF membranes. After blocked with 5% non-fat dry milk, the membranes were then incubated overnight at 4 °C with primary antibodies against: OTUB1 (Abcam, USA), TPM1, ACTA2, ATP5F1B, MDH2, RUNX2, OSX (all from Proteintech, China), DMP1 (Affinity, China), DSPP (Santa Cruz Biotechnology, USA), and GAPDH (loading control; Proteintech, China). After incubation with an HRP-conjugated secondary antibody, protein bands were visualized, and their intensities were quantified using ImageJ software. The biological replicates and their corresponding quantification were represented in Figure S4.

For Co-IP assays, DPSCs were washed with PBS and lysed on ice in 0.3% 1 × Lysis/Wash Buffer with protease inhibitors. Total protein concentration in the lysates was quantified by BCA assay for input normalization. Lysates were incubated for 2 h at 4 °C with rProtein A/G MagPoly beads (ACE Biotechnology, China) pre-coated with 5 μg of IgG (control), anti-OTUB1, or anti-TPM1 antibody. Following washing, the immunoprecipitated complexes were eluted in 1 × SDS-PAGE loading buffer and analyzed by western blotting.

### Alkaline phosphatase (ALP) staining and activity assay

ALP activity in transfected DPSCs was assessed through both histochemical staining and quantitative measurement. For staining, cells were fixed with 4% PFA for 30 min and then incubated with the NBT/BCIP substrate (Jiancheng, Nanjing) at 37 °C for 30 min according to the kit protocol. For quantitative analysis, ALP activity was measured using a commercial assay kit (Beyotime, China). The total protein concentration of each sample was determined in parallel using a BCA Protein Assay Kit, and the ALP activity values were normalized to the corresponding protein content.

### Immunofluorescence staining

Transfected DPSCs were placed in 96-well plates and cultured for 24 hours. Following this, the cells underwent washing with PBS, fixation using 4% PFA for 30 minutes, and permeabilization with 0.05% Triton X-100 (Beyotime, China) for 20 minutes. The cells were blocked using normal goat serum, incubated overnight at 4°C with primary antibodies, and then exposed to fluorochrome-conjugated secondary antibodies (Invitrogen, USA) for an hour in the dark. Images for immunofluorescence were captured with an inverted fluorescence microscope from Leica, Germany.

### Protein half-life assay

After being treated with cycloheximide from MedChemExpress, USA, the transfected DPSCs were collected at specified intervals (0, 2, 4, and 6 hours). The cell lysates were then prepared and analyzed using western blotting to evaluate protein stability.

### Proteomics analysis

Lc-Bio (Hangzhou) conducted the quantitative proteomics analysis. Cells were lysed on ice for 30 minutes, followed by centrifugation to collect the supernatant. Protein concentration was measured using a BCA assay kit according to the manufacturer's instructions. Peptides for LC-MS/MS analysis were reconstituted in 0.1% formic acid, centrifuged, and introduced into a Thermo Scientific Vanquish Neo UHPLC system with an ES906 column (150 mm). Separation was performed using a binary gradient with solvent A (0.1% formic acid in water) and solvent B (80% acetonitrile) at a flow rate of 2.5 µL/min over an 8-minute linear gradient. Data were acquired in data-independent acquisition (DIA) mode and processed using DIA-NN in a library-free mode.

### Animal experiment

The animal studies were conducted with the approval of the Medical Ethics Committee of Jiangsu Medical College (SYLL-2025-711). A rat tooth pulp wound model was established based on previously described methods.^24^^,^^25^ Briefly, female Sprague Dawley rats, aged five weeks, were employed in the study. Bilateral pulp chambers in the first molars were exposed using a 1-mm diameter dental drill under water cooling conditions. Gelatin sponges impregnated with either OTUB1-overexpressing or scrambled lentiviral particles were inserted into the cavities, which were subsequently sealed with glass-ionomer cement. After a period of two weeks, the rats were euthanized, and their maxillae were harvested for Hematoxylin and eosin (HE) staining and immunohistochemical analysis.

### Statistical analysis

The experimental data were subjected to analysis utilizing SPSS version 22.0 (IBM, USA) and GraphPad Prism (GraphPad Software, USA). The results are presented as the mean ± standard deviation (SD) derived from a minimum of three independent replicates. For comparisons among multiple groups, a one-way analysis of variance (ANOVA) was employed. In the context of protein half-life assays, degradation trajectories were examined using a two-way ANOVA, with time and group as variables, and their interaction was evaluated. Tukey’s post hoc test was applied for multiple comparisons, while Student’s t-test was utilized for pairwise comparisons. Statistical significance was determined at a two-tailed *P*-value of less than .05.

## Results

### The upregulation of OTUB1 correlates with odontogenic differentiation in DPSCs

**Fig. 1:**
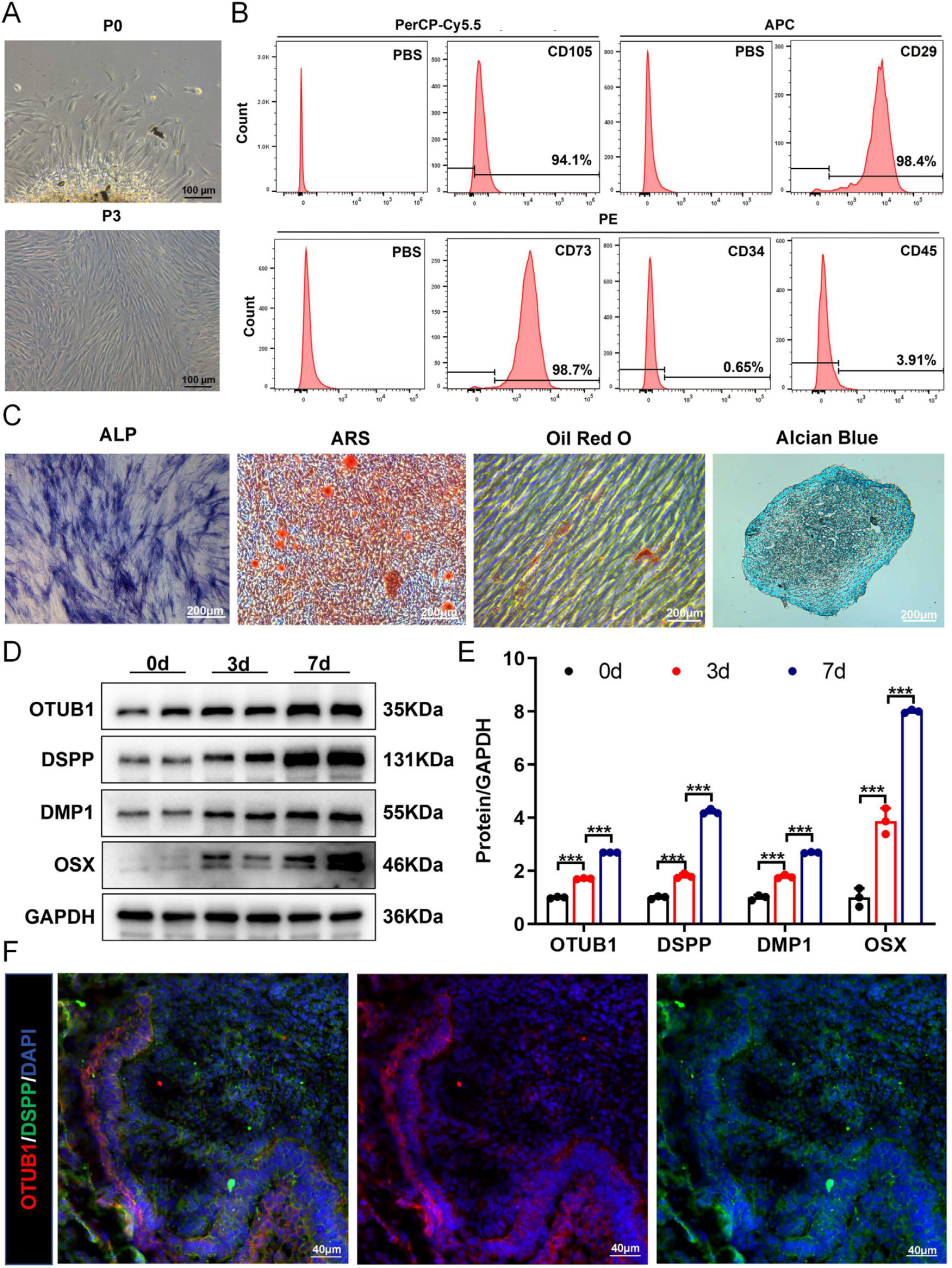
The expression of OTUB1 is positively correlated with odontogenic differentiation. A, Microscopic analysis of dental pulp stem cells (DPSCs) from both primary and third generations revealed that the DPSCs exhibited a distinct fibroblast-like morphology. B, Flow cytometric analysis demonstrated DPSCs exhibit the expression of mesenchymal stem cell markers CD29, CD73, and CD105, whereas lack hematopoietic markers CD34 and CD45. C, Results of ALP staining, ARS staining, Oil red O staining and Alcian blue staining of DPSCs showed the ability of tri-lineage differentiation of DPSCs. D and E, The expression of OTUB1 during the odontogenic differentiation of DPSCs (at days 0, 3, and 7) was analyzed by western blotting assays. F, The representative immunofluorescence staining images of molars from postnatal day 1 CD1 mice indicated the expression of OTUB1 in the odontoblast layer. n=3, * indicates *P* < .05, **indicates *P* < .01, ***indicates *P* < .001.

DPSCs were effectively isolated and displayed a characteristic fibroblast-like morphology (Figure 1A). Phenotypic characterization via flow cytometry revealed the expression of mesenchymal stem cell markers CD29, CD73, and CD105, while hematopoietic markers CD34 and CD45 were absent (Figure 1B). Under specific induction conditions, DPSCs exhibited the capacity to differentiate into osteogenic, adipogenic, and chondrogenic lineages, as evidenced by ALP, ARS, Oil Red O and Alcian blue staining, respectively (Figure 1C). Collectively, these findings collectively confirm the successful isolation of DPSCs with the potential for tri-lineage differentiation.

To assess the expression of OTUB1 during odontogenic differentiation, DPSCs were cultured in mineralization induction medium for 0, 3, and 7 days. A progressive increase in OTUB1 levels was observed throughout the mineralization period, which was concomitant with elevated expression of odontogenic markers such as DSPP, DMP1, and OSX (Figure 1D, E). Furthermore, immunofluorescence staining of 1-day-old CD1 mice molars revealed the presence of OTUB1 in the odontoblast layer (Figure 1F), suggesting its potential role in the regulation of odontogenic differentiation.

### OTUB1 enhances odontogenic differentiation in DPSCs

**Fig. 2:**
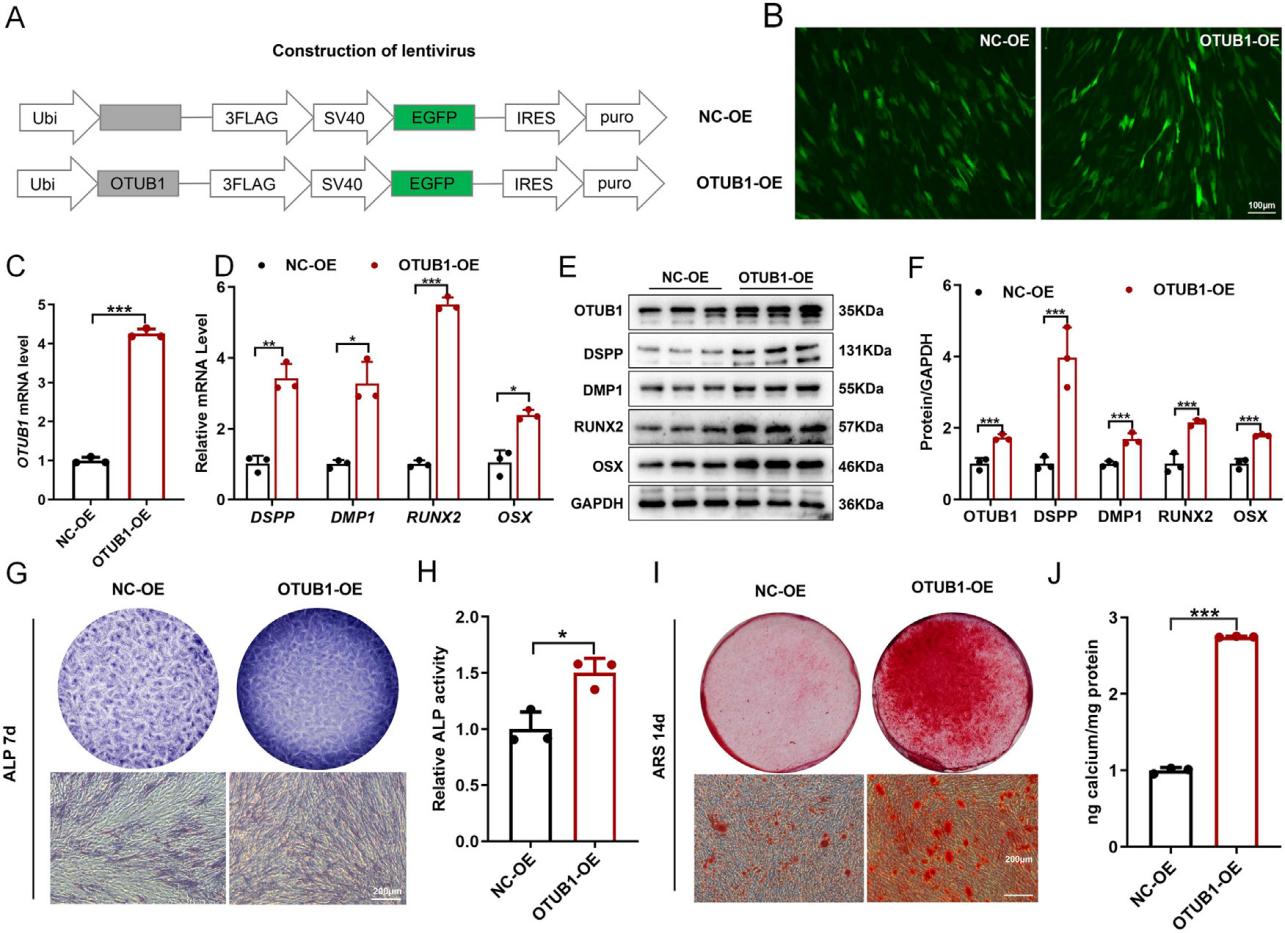
Overexpression of OTUB1 promotes the odontogenic differentiation of DPSCs. A, Schematic of lentivirus design. B and C, The transfection efficiency of OTUB1 overexpression (OTUB1-OE) and its negative control (NC-OE) in DPSCs was respectively assessed at 72 h by immunofluorescence staining and qRT‒PCR. D, qRT-PCR analysis indicated that OTUB1-OE facilitated the expression of odontogenic related genes including *DSPP, DMP1, RUNX2 and OSX*. E and F, Western blotting analysis demonstrated that OTUB1-OE increased the expression of OTUB1 and odontogenic related proteins (DSPP, DMP1, RUNX2 and OSX). G and H, Representative images of ALP staining and ALP activity analysis upon OTUB1 overexpression were exhibited in DPSCs. I and J, Representative images of ARS staining and its semi-quantification upon OTUB1 overexpression were conducted in DPSCs. n=3, * indicates *P* < .05, **indicates *P* < .01, ***indicates *P* < .001.

To investigate the effect of OTUB1 on odontoblast differentiation in vitro, we utilized a lentivirus-mediated approach to overexpress OTUB1 in DPSCs (Figure 2A). The transfection efficiency was confirmed through the proportion of fluorescent cells, qRT-PCR, and western blotting analysis (Figure 2B-F). Subsequent analyses indicated that the upregulation of OTUB1 significantly increased the mRNA and protein levels of key odontogenic markers, including DSPP, DMP1, RUNX2, and OSX (Figure 2D-F). Additionally, ALP staining/activity assays and ARS staining demonstrated enhanced matrix mineralization in DPSCs following OTUB1 overexpression (Figure 2G-J).

**Fig. 3:**
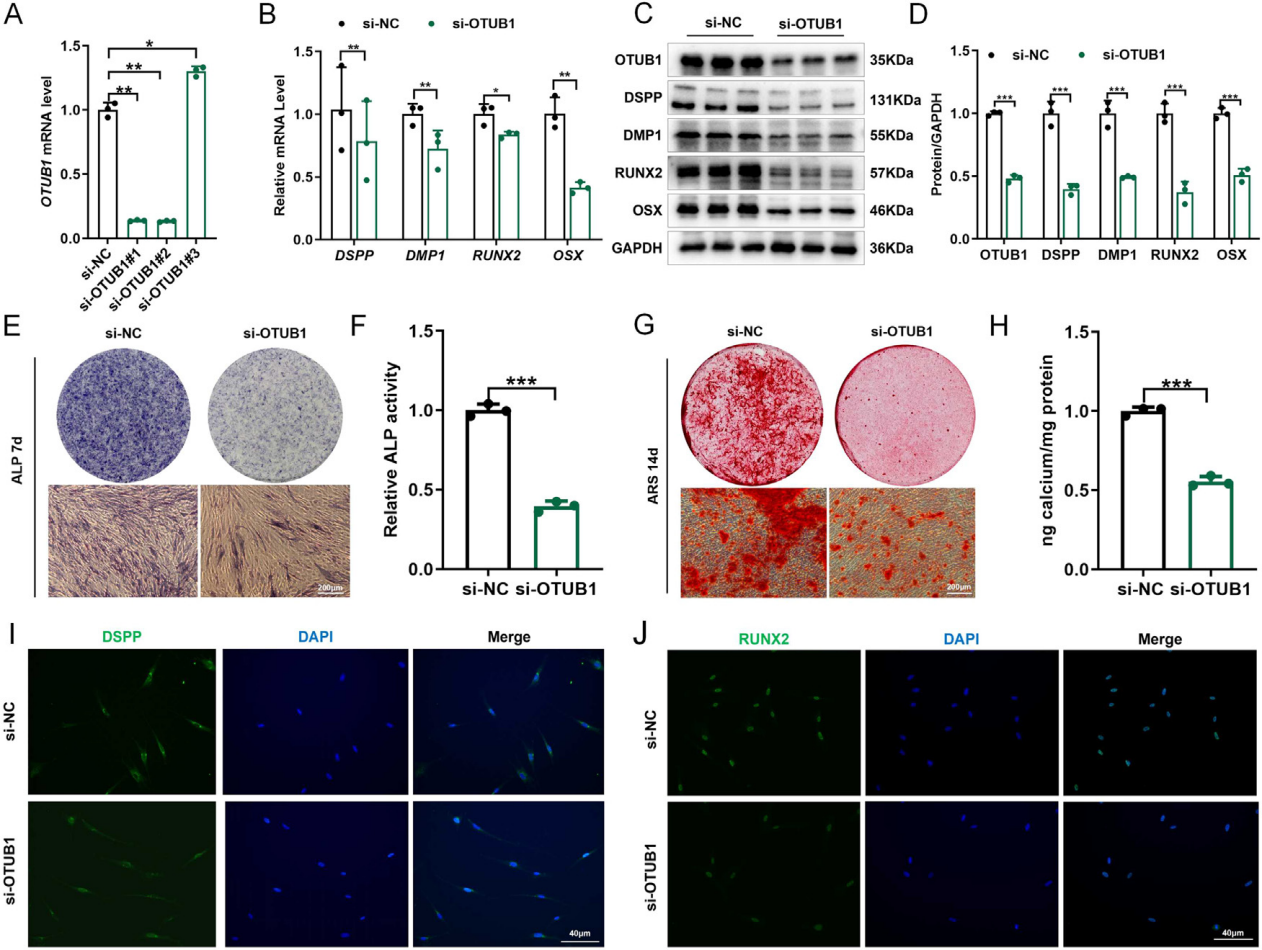
Silencing of OTUB1 inhibits the odontogenic differentiation of DPSCs. A, qRT-PCR was performed to assess the transfection efficiency following OTUB1 silencing, with si-OTUB1#2 demonstrating the highest efficacy in DPSCs. B, qRT-PCR analysis indicated that si-OTUB1 inhibited the expression of odontogenic related genes in DPSCs. C and D, Western blotting analysis showed that si-OTUB1 reduced the expression of odontogenic related proteins. E and H, Representative images of ALP staining and ARS staining in the DPSCs transfected with si-OTUB1 indicated reduced odontogenic differentiation. I and J, Immunofluorescence staining results of transfected DPSCs showed that si-OTUB1 led to a reduction in the expression levels of DSPP and RUNX2. n=3, * indicates *P* < .05, **indicates *P* < .01, ***indicates *P* < .001.

Alternatively, we applied specific siRNAs to reduce OTUB1 expression. The transfection efficiency was verified using qRT-PCR, and si-OTUB1 #2 was identified as the most potent inhibitor, thereby selected for subsequent experiments (Figure 3A). A notable reduction in the expression of odontogenic markers at both the transcript and protein levels was observed following OTUB1 knockdown (Figure 3B-D). Correspondingly, there was a marked decline in differentiation and mineralization capacity as evidenced by ALP staining/activity and ARS staining (quantified through CPC elution) (Figure 3E-H). Immunofluorescence analysis further confirmed the diminished protein expression of DSPP and RUNX2 upon OTUB1 knockdown (Figure 3I-J). Together, these findings suggest that OTUB1 functions as a positive regulator of odontogenic differentiation in DPSCs.

### OTUB1 enhances TPM1 protein stability in DPSCs by interacting with and deubiquitinating TPM1

**Fig. 4:**
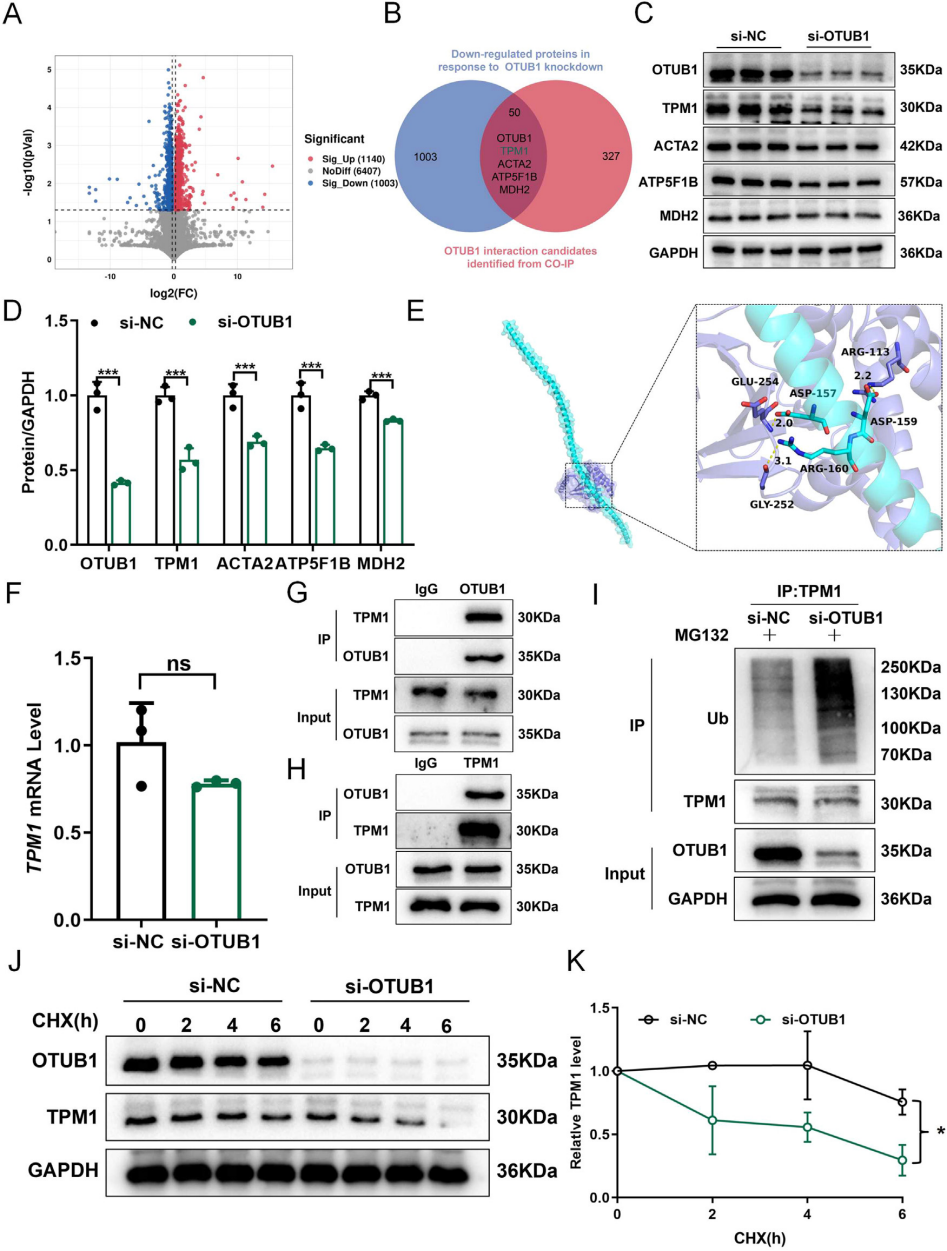
OTUB1 interacts with TPM1 and promotes TPM1 protein stability through deubiquitination in DPSCs. A, Volcano plot illustrating the differential expression of proteins (DEPs) in transfected DPSCs. B, Strategy employed for the identification of substrate proteins, identified by comparing downregulated proteins from proteomic analysis with candidate interacting proteins obtained from Co-IP experiments. C and D, Immunoblot and statistical analysis of top five DEPs were performed to screen for the target proteins upon OTUB1 silencing in DPSCs. E, Molecular docking model of OTUB1(purple) with TPM1(green). F, qRT-PCR analysis of TPM1 upon OTUB1 knock down in DPSCs. G and H, Co-IP of OTUB1 and TPM1 in DPSCs. I, Ubiquitination of TPM1 protein upon OTUB1 inhibition increased in DPSCs. J and K, Measurement of TPM1 degradation in DPSCs upon OTUB1 inhibition. n=3, * indicates *P* < .05, ***indicates *P* < .001.

OTUB1 functions by deubiquitinating and stabilizing target proteins. Therefore, we defined candidate substrates as proteins that: (1) physically interact with OTUB1, and (2) exhibit decreased steady-state levels following OTUB1 knockdown. Proteomics analysis revealed 1140 upregulated and 1003 downregulated differentially expressed proteins (DEPs) with a fold change of less than 0.83 and a *P*-value of less than .05 following OTUB1 knockdown in DPSCs (Figure 4A). Simultaneously, LC-MS/MS analysis was performed to identify target interacting proteins. The top five candidate substrates in DPSCs were prioritized (Figure 4B). Western blotting analysis was performed to validate these candidates and TPM1 revealed a significant reduction in OTUB1 knockdown DPSCs (Figure 4C, D). To investigate the potential interaction between OTUB1 and TPM1, the GRAMM web server was utilized to elucidate the characteristics and underlying mechanisms of their interactions. The resulting docking complex demonstrated favorable structural complementarity, as evidenced by a notable GRAMM docking score of −451, indicative of stable binding characteristics between OTUB1 and TPM1. Several intermolecular hydrogen bonds were identified at the interaction interface, predominantly involving OTUB1 Arg113 and TPM1 Asp159, which contributed to the stabilization of the protein–protein complex (Figure 4E). Intriguingly, the regulation of TPM1 expression by OTUB1 occurred exclusively at the protein level, with no changes in its mRNA level, indicating that OTUB1 might be involved in the posttranslational modification of TPM1(Figure 4F). CO-IP analysis on DPSCs verified that TPM1 was present in the anti-OTUB1 immunoprecipitate, but absent in the immunoglobulin G (IgG) immunoprecipitate from the cell lysate (Figure 4G). In a similar manner, OTUB1 was identified in the anti-TPM1 immunoprecipitate, suggesting the presence of an interaction between endogenous OTUB1 and TPM1(Figure 4H). Additionally, the elimination of endogenous OTUB1 led to a notable rise in the polyubiquitination of TPM1 (Figure 4I). These data demonstrate that OTUB1 stabilizes TPM1 by means of direct deubiquitination, thereby preventing its proteasomal degradation. Afterwards, we treated DPSCs with cycloheximide (CHX), one kind of translational inhibitor, to monitor the half-life of TPM1 protein upon OTUB1 knockdown (Figure 4J, K). The result indicated that the half-life of TPM1 was markedly reduced upon OTUB1 knockdown, confirming that OTUB1 is required to maintain TPM1 protein stability.

**Fig. 5:**
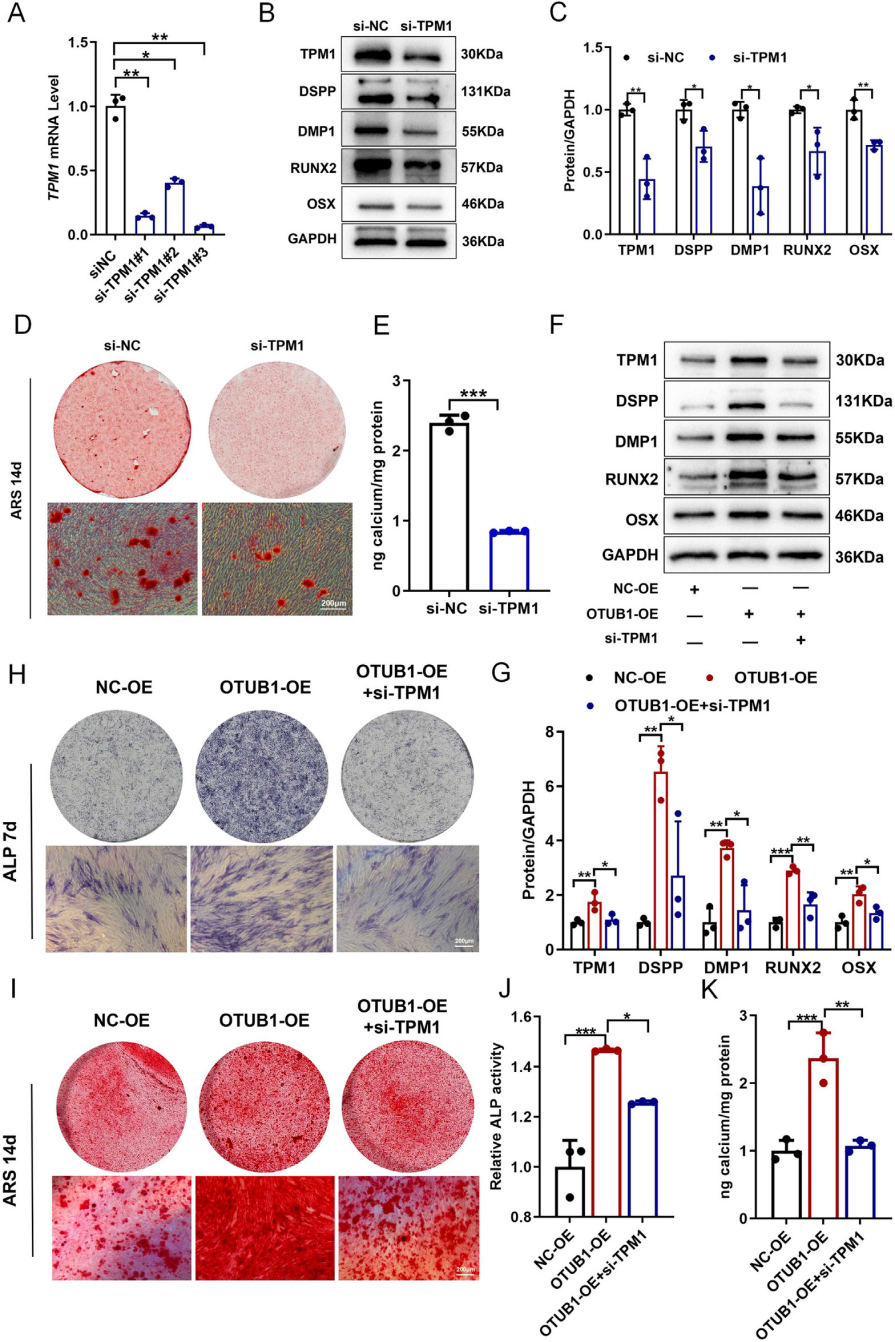
OTUB1 mediated deubiquitination regulates the odontogenic differentiation of DPSCs via TPM1 stability. A, qRT-PCR confirmed the effective knockdown of TPM1 in DPSCs. B and C, Reducing TPM1 levels led to a decrease of TPM1 and odontogenic protein expression in DPSCs. D and E, Representative images of ARS staining and quantification demonstrated that si-TPM1 decreased odontogenic differentiation in DPSCs. F and G, The promotion of odontogenic protein expression caused by OTUB1-OE was inhibited by cotransfection of DPSCs with si-TPM1. H and K, ARS staining and ALP staining demonstrated that the increasing in calcium nodules induced by OTUB1-OE was abrogated by cotransfection with si-TPM1. n=3, * indicates *P* < .05, **indicates *P* < .01, ***indicates *P* < .001.

To ascertain the role of OTUB1 in regulating the odontogenic differentiation of DPSCs through the mediation of TPM1 deubiquitination, we conducted an in-depth analysis of the influence exerted by TPM1 on the odontogenic differentiation process of DPSCs. A transient transfection procedure utilizing si-TPM1 was performed (Figure 5A). The findings from western blotting analysis and ARS staining indicated that the suppression of TPM1 expression impedes the odontogenic differentiation trajectory of DPSCs (Figure 5B-E). Moreover, the elevated levels of TPM1 and differentiation markers resulting from OTUB1 overexpression were successfully reversed through co-transfection with si-TPM1 (Figure 5F, G). This functional restoration was additionally validated by ALP, ARS staining and their respective quantitative analyses (Figure 5H-K). However, the capacity of odontogenic differentiation remained unaffected by si-NC when co-transfected with OTUB1-OE in DPSCs (Figure S1).

### OTUB1 enhances odontogenic differentiation in DPSCs by activating autophagy

**Fig. 6:**
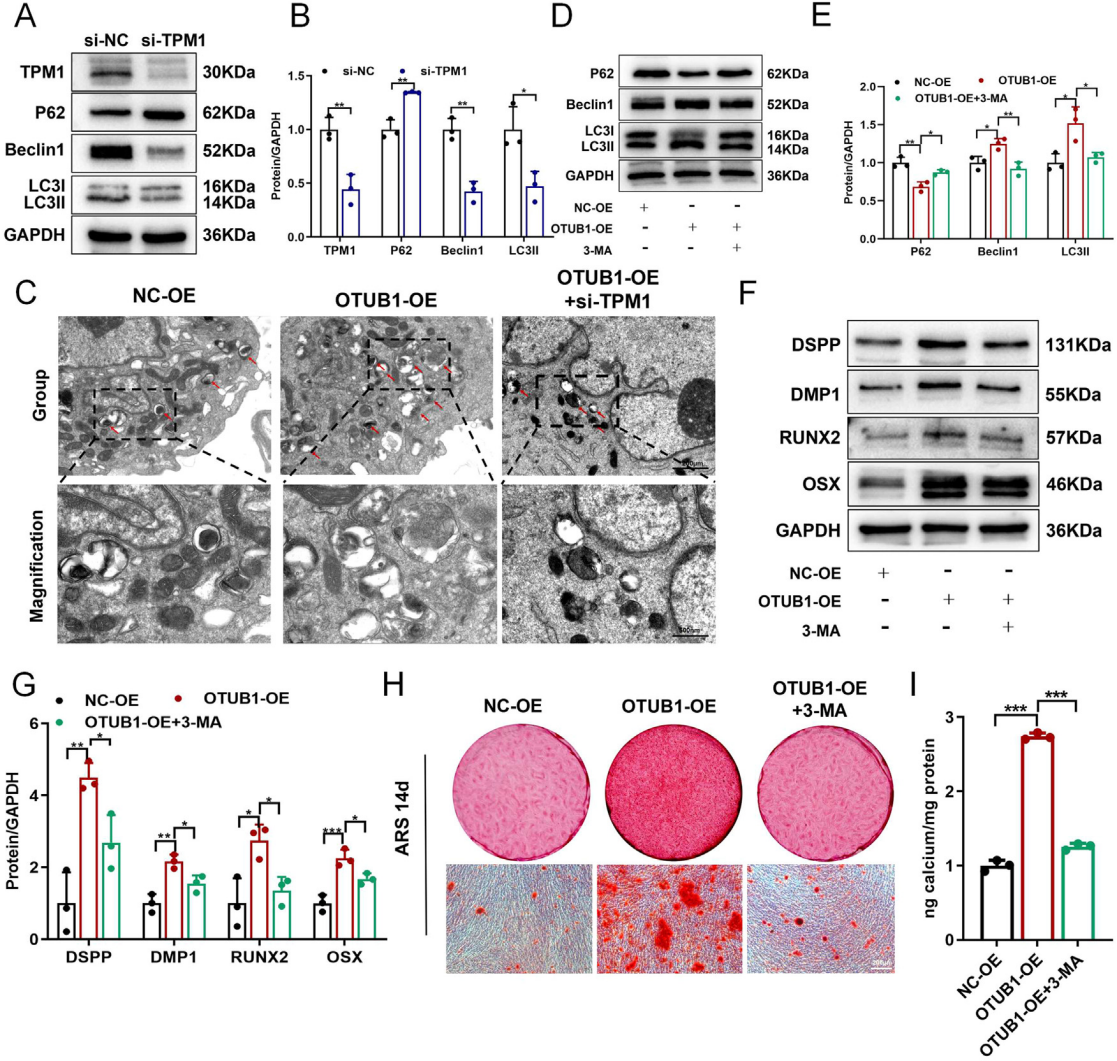
OTUB1/TPM1 axis induces odontogenic differentiation by activating autophagy. A and B, Inhibition of TPM1 decreased the expression of autophagy-related proteins in DPSCs. C, TEM analysis showed the number of autophagolysosomes production increased after OTUB1 overexpression, while the application of si-TPM1 in conjunction with OTUB1 overexpression reversed this trend in DPSCs. Autophagolysosomes are denoted by red arrows in the images. D and E, The enhancement of autophagy-related protein expression by OTUB1 overexpression was abolished upon co-treatment with the autophagy inhibitor 3-MA. F and G, 3-MA downregulated the expression of odontogenic protein markers in DPSCs in the presence of OTUB1-OE. H and I, ARS staining and its quantification analysis illustrated that 3-MA slightly reversed the generation of mineralized nodules increased by OTUB1-OE in DPSCs. n=3, * indicates *P* < .05, **indicates *P* < .01, ***indicates *P* < .001.

Research has demonstrated that TPM1 is associated with autophagy,^26^ a critical mechanism for regulating the odontogenesis process in DPSCs.^27^^,^^28^ Nevertheless, the involvement of autophagy in the differentiation of DPSCs mediated by the OTUB1/TPM1 axis has yet to be explored. As expected, the western blotting analysis demonstrated that the suppression of TPM1 led to diminished autophagic activity (Figure 6A, B). In parallel, rapamycin,^28^ a well-recognized inducer of autophagy, was administered to TPM1 knockdown DPSCs. The findings indicated that rapamycin effectively reversed the diminished autophagic capacity observed in si-TPM1 treated cells (Figure S2), which demonstrated the potential role of TPM1/autophagy in DPSCs. Furthermore, transmission electron microscopy (TEM) images revealed an increased presence of autophagosomes in OTUB1-overexpressing DPSCs compared to control DPSCs. Conversely, the application of si-TPM1 resulted in a reduction of autophagosomes in OTUB1-overexpressing DPSCs (Figure 6C). Additionally, the elevated levels of autophagy-related proteins associated with OTUB1 overexpression were significantly attenuated by co-transfection with 3-MA (Figure 6D, E). Taken together, these findings suggest that OTUB1 overexpression facilitates the activation of the autophagy process via its interaction with TPM1.

To further evaluate the influence of autophagy on odontogenesis in DPSCs, we employed 3-methyladenine (3-MA) and chloroquine (CQ), pharmacological agents known for its specific inhibition of the autophagic process.^28^, ^29^, ^30^ The results of the western blotting assay indicated that the enhancement of odontogenesis in DPSCs induced by OTUB1 overexpression was inhibited by co-stimulation with 3-MA (Figure 6F, G), which is consistent with the outcomes observed in the ARS staining analysis (Figure 6H, I). Similarly, CQ was observed to attenuate the enhanced odontogenic differentiation of DPSCs with overexpression of OTUB1 (Figure S3). Conversely, the solvent used for 3-MA and CQ, DMSO, did not demonstrate a significant impact on the odontogenic differentiation of DPSCs within the OTUB1-OE groups (Figure S1). Consequently, our findings demonstrate that the OTUB1/TPM1 axis plays a pivotal role in modulating the odontogenic differentiation of DPSCs by activating autophagic pathways.

### OTUB1 regulates the odontogenesis of DPSCs in vivo

**Fig. 7:**
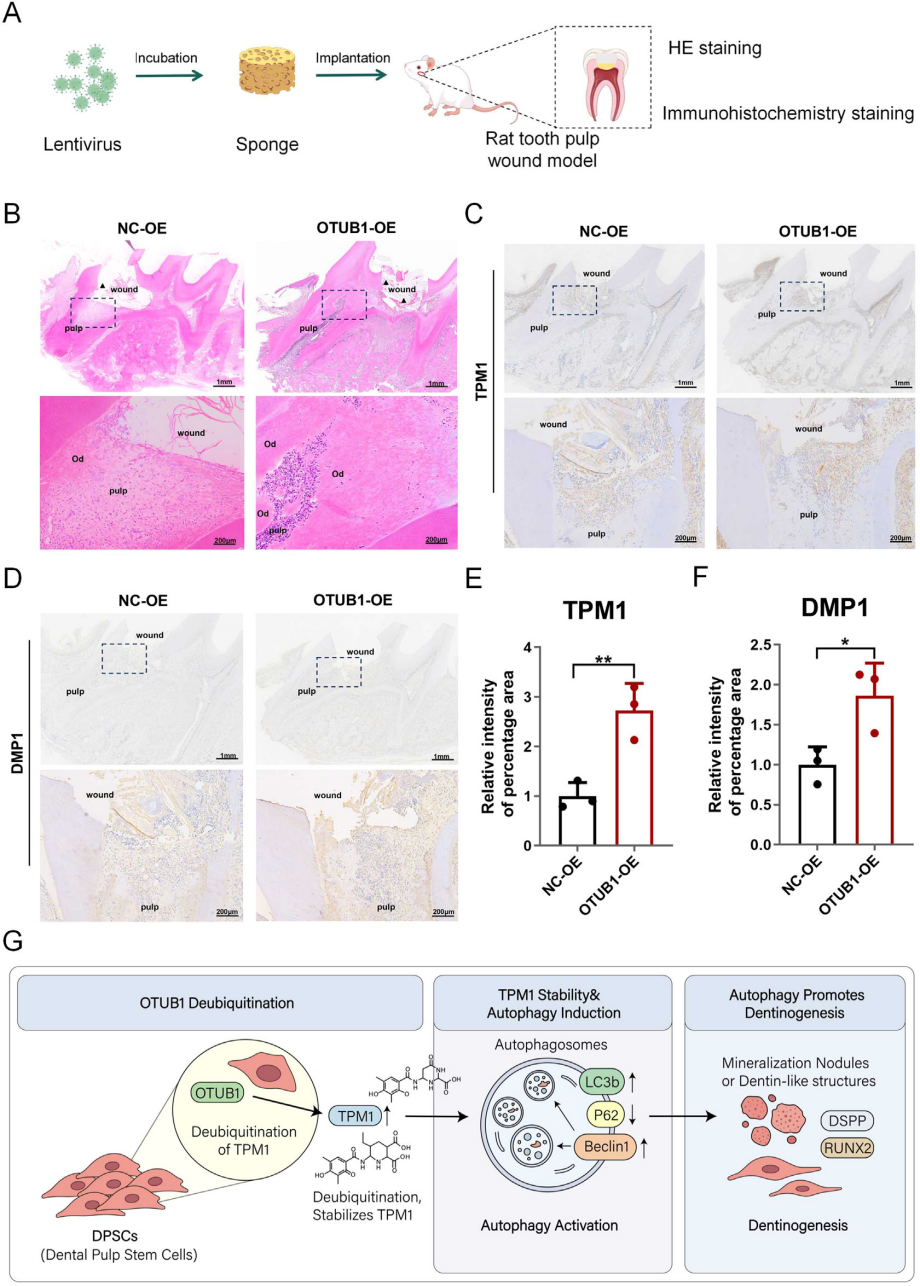
OTUB1 promotes dentin regeneration in vivo. A, A schematic representation of the rat tooth pulp wound model was created using Figdraw. B, Representative HE analyses of rat molars treated with OTUB1-OE lentivirus or subjected to scramble stimulation demonstrated a significant increase in reparative dentin formation in the group overexpressing OTUB1 compared to the control group. Od: osteodentin; Black arrow: residual glass-ionomer cement. C and F, Immunohistochemical staining analysis of TPM1, DMP1 in the defect region. G, The mechanism diagram of OTUB1 facilitating the odontogenic differentiation of DPSCs. n=3, * indicates *P* < .05, **indicates *P* < .01.

To assess the in vivo odontogenic regulatory function of OTUB1, lentiviral particles engineered for OTUB1 overexpression or containing a scrambled control sequence were incorporated into gelatin sponge scaffolds. These scaffolds were subsequently inserted into 1 mm dental pulp defects in SD rats. Two weeks following the treatment, the samples were collected for subsequent analysis (Figure 7A). The results of HE staining revealed that the OTUB1 group demonstrated significant reparative dentin formation compared to the control group (Figure 7B). Immunohistochemical staining further confirmed elevated TPM1 expression in the OTUB1-overexpressing group relative to the control (Figure 7C, E). Finally, increased DMP1 expression was also detected in the OTUB1-overexpressing group (Figure 7D, F), supporting that OTUB1 promotes the odontogenesis of DPSCs through deubiquitination of TPM1.

As illustrated in the Graphical Abstract, the upregulation of OTUB1 significantly enhanced the odontogenic differentiation capacity of DPSCs. Mechanistically, OTUB1 facilitated the deubiquitination of TPM1, subsequently activating autophagy, a critical process in dentin regeneration (Figure 7G).

## Discussion

Stem cells are highly promising for regenerative medicine because of their capacity to differentiate into various cell types. DPSCs, derived from dental pulp tissue, are particularly notable for their high proliferative potential and multidirectional differentiation capacity – key properties that make them highly suitable for pulp regeneration.^8^ Additionally, due to their minimally invasive harvesting from discarded teeth, low immunogenicity, and lack of ethical concerns, DPSCs have emerged as a uniquely attractive cell source for clinical applications.^31^, ^32^, ^33^ Notably, Liu et al have developed an allogeneic human DPSC injection for the treatment of periodontitis, which is currently under clinical trial.^34^ Our study further investigated the role of DPSCs in pulp regeneration and explores the underlying mechanisms involved.

Deubiquitination, a reversible post-translational modification, involves the catalytic cleavage of single ubiquitin (Ub) molecules or polyubiquitin chains from proteins. This process is facilitated by six distinct families of enzymes: ubiquitin-specific proteases (USPs), ubiquitin C-terminal hydrolases (UCHs), ovarian tumor proteases (OTUs), Josephins, the JAB1/MPN/MOV34 family (JAMMs), and the motif interacting with Ub-containing novel deubiquitinating enzyme family (MINDYs).^35^^,^^36^ Deubiquitination counteracts the ubiquitin cascade by inhibiting the degradation of proteins conjugated to a ubiquitin chain. This process is essential for maintaining protein homeostasis, modulating protein stability, and regulating diverse signaling pathways.^37^ Among them, OTUB1, which is derived from OTU domain family of DUBs,^17^ was reported its significant function in the regulation of stem cells. For instance, OTUB1 has revealed its pivotal role in maintaining bone homeostasis by stabilizing fibroblast growth factor receptor 2 (FGFR2), a key regulator of osteogenesis, underscoring the therapeutic potential of targeting OTUB1 to alleviate conditions such as osteoporosis.^22^ Wang et al developed a novel class of deubiquitinase-targeting chimeras, designated as HY-X3369, and demonstrated that OTUB1 plays a critical role in mediating the osteogenic effects of HY-X3369 in BMSCs.^38^ Our study revealed the novel role of OTUB1 mediated deubiquitination in the process of odontogenic differentiation of DPSCs.

TPM1, a member of the tropomyosin family, is an actin-binding protein that plays a crucial role in the regulation of the actin cytoskeleton, which is fundamental to various cellular processes and tissue development.^39^^,^^40^ Prior studies have elucidated the potential involvement of TPM1 in the differentiation processes of MSCs. Brielle et al provided evidence that TPM1 facilitates the nuclear localization of YAP1, thereby promoting osteogenic differentiation in MSCs, as revealed through single-cell RNA sequencing analysis.^41^ Additionally, Wang et al observed that YO-EVs mediate the delivery of TPM1 protein to BMSCs, leading to increased matrix stiffness and elevated expression of osteogenic markers.^42^ Nevertheless, the prospective role of TPM1 in regulating the differentiation of dental-derived stem cells, as well as the deubiquitination of TPM1 through posttranslational modification, remains unknown. In this study, TPM1 was identified as a potential target via proteomics analysis, which was subsequently validated using CO-IP and gain- and loss-of-function analysis. Our findings elucidated the role of OTUB1/TPM1 signaling pathways in the committed differentiation of DPSCs.

Autophagy is defined as an evolutionarily conserved catabolic process whereby cells degrade damaged components and recycle biomolecules to sustain homeostasis. Beyond this essential housekeeping role, it acts as a critical regulator of diverse cellular functions – notably energy balance – and broader physiological processes including aging.^43^, ^44^, ^45^, ^46^ Dysfunctional autophagy contributes to the pathogenesis of various age-related diseases, including neurodegenerative disorders^47^ and cancer.^48^ Emerging findings emphasize the pivotal role of autophagy as a central regulator in the physiological and pathological processes of dental-derived stem cells. Wei et al have notably shown that serum starvation influences autophagy in human periodontal ligament cells through a reactive oxygen species-mediated AMPK/mTOR signaling pathway, highlighting the critical role of redox and metabolic signaling in regulating autophagic activity within dental-derived cell populations.^49^ Our previous study demonstrated that Circ-ZNF236 was involved in controlling the odontogenic differentiation of stem cells from the apical papilla (SCAPs) through the activation of autophagy.^30^ In addition to autophagy, alternative forms of programmed cell death, such as pyroptosis, have been implicated in dental inflammatory and tissue remodeling processes. For example, Chen et al demonstrated that HDAC9-mediated pyroptosis exacerbates orthodontically induced inflammatory root resorption, thereby uncovering a crucial epigenetic mechanism that connects inflammatory cell death to pathological hard tissue resorption within the dentoalveolar complex.^50^ Collectively, these findings underscore the notion that multiple regulated cell death and recycling pathways collaboratively regulate dental tissue homeostasis, differentiation, and disease progression. Our research further elucidated the connection between deubiquitination and autophagy in the context of odontogenic differentiation of DPSCs. In this study, we found that TPM1 modulates the autophagy pathway, as evidenced by experiments with both gain and loss function. To determine whether autophagy mediates the pro-odontogenic effect of OTUB1, we assessed key differentiation markers in OTUB1-overexpressing DPSCs with or without the autophagy inhibitor 3-MA and CQ. The results indicated that 3-MA or CQ treatment attenuated the odontogenic capacity of OTUB1-overexpressing DPSCs. These findings provide novel mechanistic insights into how deubiquitination and autophagy jointly regulate DPSC odontogenesis.

## Conclusion

In conclusion, our findings elucidate a mechanism whereby OTUB1 regulates DPSC differentiation via TPM1 deubiquitination and autophagy modulation. This highlights the translational potential of targeting OTUB1 to intervene in and promote dentin regeneration.

## Ethics approval and consent to participate

This research adhered to all applicable ethical guidelines. The Medical Ethics Committee of Jiangsu Medical College (SYLL-2025-711) approved the animal procedures. Human tooth sample collection was conducted following a protocol approved by the Ethics Committee of Yancheng Stomatological Hospital (PJ2024-022-01), with all donors providing informed consent.

## Availability of data and materials

The data sets that back up the findings of this article can be obtained from the corresponding author upon a reasonable request, adhering to ethical standards and confidentiality agreements with participants.

## Funding

This research was financially supported by the Yancheng Science and Technology Bureau through the Applied Basic Research grant (YCBK2025046) and the Key R&D Program (YCBE202428). Additional funding was provided by the School-Local Collaborative Innovation Project of Jiangsu Medical College (grant numbers 202491408 and 202591401).

## Author contributions

*Drafted the manuscript:* Tingjie Gu, Wei Song. *Responsible for the experimental design*: Dandan Song, Rui Gu, Qiang Chen. *Collected and analyzed the data*: Yifan Pan, Jie Yi. *Contributed to the revision of the manuscript*: Tao Xu, Kai Xu, Zhengmei Qian.

## Conflict of interest

None disclosed.

## Appendix Supplementary materials

Image, application 1

## Footnotes

Supplementary material associated with this article can be found in the online version at doi:10.1016/j.identj.2026.109814.
