# Supplementary material for: OTUB1 Promotes Odontogenesis in Dental Pulp Stem Cells by Mediating TPM1 Deubiquitination and Activating Autophagy
Source: Int Dent J. 2026 Aug 6;76(5):109814. doi: 10.1016/j.identj.2026.109814 (PMC13475200; doi:10.1016/j.identj.2026.109814)
Supplement: Supplementary file 1 [file mmc1.docx]

**OTUB1 promotes odontogenesis in dental pulp stem cells by mediating TPM1 deubiquitination and activating autophagy**

**Authors: Tingjie Gu^a*^, Wei Song^a，b*^, Yifan Pan^a^, Jie Yi^a^, Tao Xu^c^, Kai Xu^a^, Zhengmei Qian^a^, Dandan Song^a#^, Rui Gu^a#^, Qiang Chen^a#^**

^a^ Yancheng Stomatological Hospital, No. 143, Jiefang South Road, Yancheng, Jiangsu, China

^b^ Xuzhou Medical University, Xuzhou, Jiangsu, China

^c^ Department of Stomatology, Nanjing Central Hospital, Nanjing, Jiangsu, China

* Tingjie Gu and Wei Song have made equal contributions to this manuscript.

\# The authors shared equal contributions to this work.

**# Corresponding Authors:**

Email: [1109726757@qq.com (Dandan](mailto:1109726757@qq.com%20(Dandan) Song) [458297927@qq.com (Rui](mailto:458297927@qq.com%20(Rui) Gu) kqyytxdz2024@163.com (Qiang Chen)

**Supplementary Figures**

Fig. S1 The analysis of OTUB1-OE+vehicle group. (A-B) The results of Western blotting analysis demonstrated that neither OTUB1-OE+si-NC nor OTUB1-OE+DMSO significantly affected the odontogenic potential of DPSCs. (C-F) ALP staining and ARS staining, along with their quantification analysis indicated no significant formation of mineralized nodules among the three groups of DPSCs. n=3, ns indicates no statistical significance.

Fig.S2 Rapamycin reversed the inhibition of proteins related to autophagy. n=3, * indicates *P*<0.05, **indicates *P*<0.01, ***indicates *P*<0.001.

Fig.S3 The promotion of odontogenic differentiation through OTUB1 overexpression was suppressed by CQ treatment. (A-B) Protein expression levels were assessed in NC-OE, OTUB1-OE, and OTUB1-OE+CQ groups within DPSCs. (C) Representative images of ALP staining were provided. n=3, * indicates *P*<0.05, **indicates *P*<0.01.

Fig.S4 Biological replicates utilized in the western blotting assay are presented. Replicate (A) corresponds to Figure 4J, replicate (B) corresponds to Figure 5B, replicate (C) corresponds to Figure 5F, replicate (D) corresponds to Figure 6A, replicate (E) corresponds to Figure 6D, and replicate (F) corresponds to Figure 6E.

**Supplementary Tables**

| **The oligonucleotide name** | **Sense sequences (5'-3')** |
| --- | --- |
| si-OTUB1 1 | CCGACTACCTTGTGGTCTA |
| si-OTUB1 2 | GACACTACGATATCCTCTA |
| si-OTUB1 3 | GCAGGACCGAATTCAGCAA |
| si-TPM1 1 | CGGAGAGGTCAGTAACTAA |
| si-TPM1 2 | CGCAGAAGGAAGACAGATA |
| si-TPM1 3 | CATTGAGAGTCGAGCCCAA |

Supplemental Table 1. The oligonucleotide sequences used in the study.

| **Gene** | **Forward primers (5’-3’)** | **Reverse primers (5’-3’)** |
| --- | --- | --- |
| *OTUB1* | TCGGTCCTATACAAGGAGTATGC | GGTCTTGCGGATGTACGAGT |
| *TPM1* | GCCGACGTAGCTTCTCTGAAC | TTTGGGCTCGACTCTCAATGA |
| *DSPP* | ATATTGAGGGCTGGAATGGGGA | TTTGTGGCTCCAGCATTGTCA |
| *DMP1* | AGGAAGTCTCGCATCTCAGAG | TGGAGTTGCTGTTTTCTGTAGAG |
| *RUNX2* | TCTTAGAACAAATTCTGCCCTTT | TGCTTTGGTCTTGAAATCACA |
| *OSX* | CCTCCTCAGCTCACCTTCTC | GTTGGGAGCCCAAATAGAAA |
| *GAPDH* | GGAGCGAGATCCCTCCAAAAT | GGCTGTTGTCATACTTCTCATGG |

Supplementary Table 2. The primer sequences used for real-time PCR.
